# The new clinical classification of metastatic spinal malignancies serves as a vital reference for surgical management: a retrospective case-control study

**DOI:** 10.1186/s12891-023-07092-4

**Published:** 2023-12-08

**Authors:** Junjun Bai, Jian Li, Jia Lv, Wangzhe Yang, Yushan Wang, Yi Feng, Zhi Lv

**Affiliations:** 1 Department of Orthopaedics, Second Hospital of Shanxi Medical University, Taiyuan, 030001 China; 2 Department of Orthopaedics, Third Hospital of Shanxi Medical University, Shanxi Bethune Hospital, Shanxi Academy of Medical Sciences, Tongji Shanxi Hospital, Taiyuan, 030032 China

**Keywords:** Metastatic spinal malignancies, Clinical classification, Offending arterial embolization, Surgical intervention

## Abstract

**Background:**

It is commonly accepted that surgical treatment is an essential component of the comprehensive management of metastatic spinal malignancies. However, up until now, the clinical classification of metastatic spinal malignancies has not been well-structured.

**Methods:**

After IRB approval, 86 patients with metastatic spinal malignancies were adopted. According to the vascular distribution, stability of vertebrae, and degree of nerve compression, metastatic spinal malignancies can be classified into five types. Tumors classified as type I typically appear in the vertebral body. Type II tumors are those that develop in the transverse processes, superior and inferior articular processes, and spinal pedicles. Type III denotes malignancies that are present in the spinous process and vertebral plate. Types IVa and IVb are included in type IV. Type IVa combines type I and type II, whereas type IVb combines type II and type III. Type V tumors are those of types I, II, and III that co-occur and spread in different directions into the spinal canal. 20 of included 86 patients who did not receive segmental arterial embolization were set as the non-embolization group. The embolization group included 24 patients who received segmental arterial embolization on both sides of the diseased vertebrae. 42 patients were included in the offending embolization group after receiving responsible arterial embolization. A surgical intervention was performed within 24 h following an embolization. Surgical intervention with the purpose of removing as much of the tumor as possible and providing an effective reconstruction of the spinal column.

**Results:**

In comparison with the non-embolization group and embolization group, the offending embolization group presented unique advantages in terms of bleeding volume (p<0.001), operation time (p<0.001), and local recurrence rate within 12 months (p=0.006).

**Conclusion:**

By significantly reducing surgical trauma and local recurrence rate (12 months), responsible arterial vascular embolization procedures together with associated surgical protocols developed on the basis of the clinical classification of metastatic spinal malignancies, are worthy of clinical dissemination.

## Introduction

During the late 20th and early 21st centuries, global demographics indicated a clear trend towards an aging population. Age significantly increases the risk of chronic illnesses, including various types of tumours [1]. Several kinds of tumors can easily metastasize to the spine through hematogenous spread, causing metastatic spinal malignancies [2]. In accordance to some reports, approximately 30% of patients with tumors developed spinal metastases [3, 4]. Furthermore, this percentage is predicted to grow over time as a result of technological advancements that have significantly extended their lives and improved their treatment outcomes [5]. Generally speaking, local hematogenous dissemination, lymphatic dissemination, or implantation metastases are no longer present in metastatic spinal malignancies, which are typically locally infiltrative in growth [6]. Metastatic spinal malignancies are commonly osteolytic, destabilizing the spine and compressing the spinal cord and nerve roots [7]. The purpose of the surgical procedure is to relieve pain, reestablish spinal stability, release the compression of the spinal cord and nerves, and quickly help patients regain their self-care abilities [8].

As we all know, the Enneking, Tomita, and WBB (Weinstein-Boriani-Biagini) scores can often offer surgical therapy guidelines for primary spinal tumors [9, 10]. In recent years, several new scoring systems have been developed to guide surgical treatment strategies and prognosis in patients with metastatic spinal malignancies. The H_2_-FAILS score created by Musharbash accurately predicts 30-day mortality after surgery for metastatic spinal malignancies [11]. The SINS (Spinal Instability Neoplastic Score) score, according to Chen et al., may be employed to evaluate spinal column stability in patients with metastatic spinal malignancies [12]. The ESCC (Epidural Spinal Cord Compression) scale can be used to assess the level of spinal cord compression brought on by metastatic spinal malignancies [13]. However, although researchers have dedicated the past few decades to developing new scoring and classification systems for metastatic spine malignancies, the specific spatial classification of metastatic spinal malignancies has not been described.

Clinically, physicians may choose to preoperatively angiograph tumor supply arteries in metastatic spinal malignancies to perform vascular embolization at the relevant blood supply sites. Furthermore, preoperative imaging of the tumor supply arteries may assist the physician in assessing the level of tumor erosion, as hematogenous spread is the primary pathway by which the primary tumor metastasizes to the vertebral body. In this study, to help surgeons make surgical decisions, a specific clinical classification was constructed based on the characteristics of the blood supply to metastatic spinal malignancies. Also, the potential of employing the classification to guide surgical plans is evaluated. Based on the assessment of effective interventions and therapy outcomes in the included patients, in other words, this study is also a proposal of a new concept in the treatment of metastatic spinal malignancies.

## Materials and methods

### Patients

The clinical and radiological data of patients with metastatic spinal tumors who underwent surgical treatment in the Second Hospital of Shanxi Medical University from January 2014 to March 2020 were collected for this retrospective analysis. 86 patients were finally included in this study. The general systemic condition, degree of spinal cord compression (ESCC score), spinal instability (SINS score), and clinical classification of patients were assessed to determine the surgical intervention.

Patients who met the following criteria were included: (1) patients pathologically diagnosed with metastatic spinal malignancies, (2) patients with solitary spinal metastasis in the thoracic or lumbar vertebrae, and (3) patients treated with preoperative angiography, embolization, and reconstructive surgery. The exclusion criteria were: (1) patients who had incomplete imaging examination of the invaded vertebral body before operation, (2) patients with metastatic spinal malignancies that were not treated by the preoperative angiography and embolization, and (3) patients whose tumors were found to have blood supply by the AKA (Artery of Adamkiewicz) during embolization.

### Vertebral zone division of metastatic spinal malignancies

**Fig. 1:**
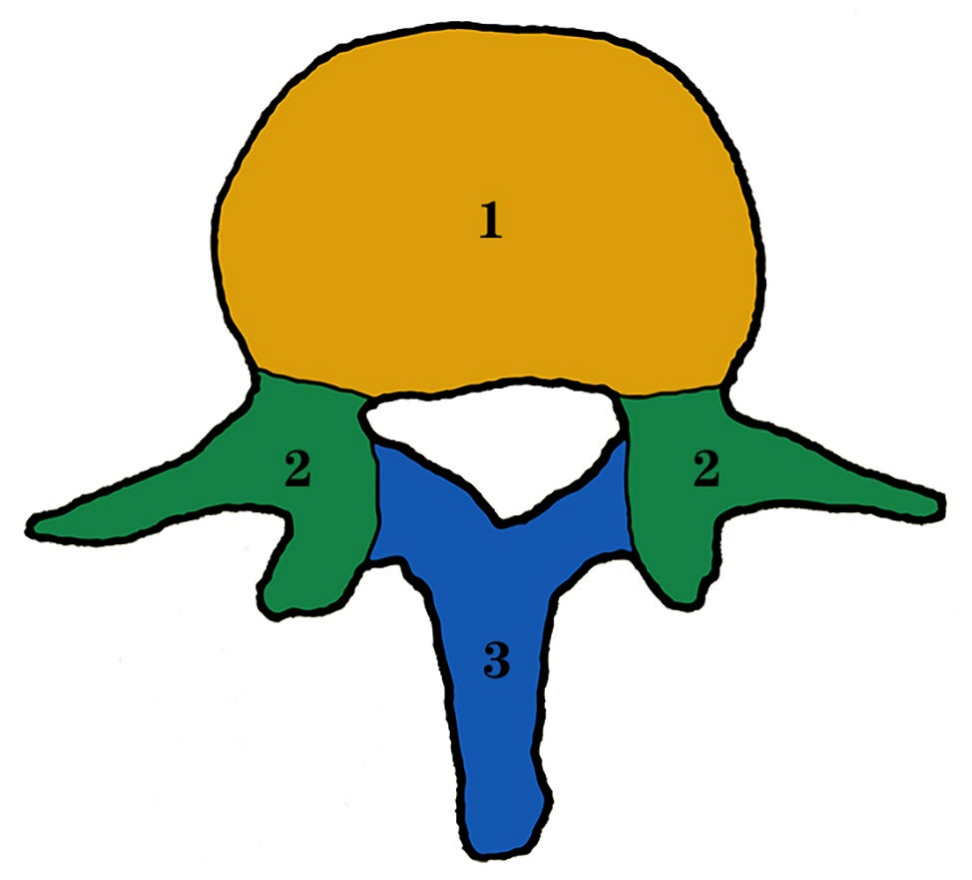
Vertebral zone division in transverse section

**Fig. 2:**
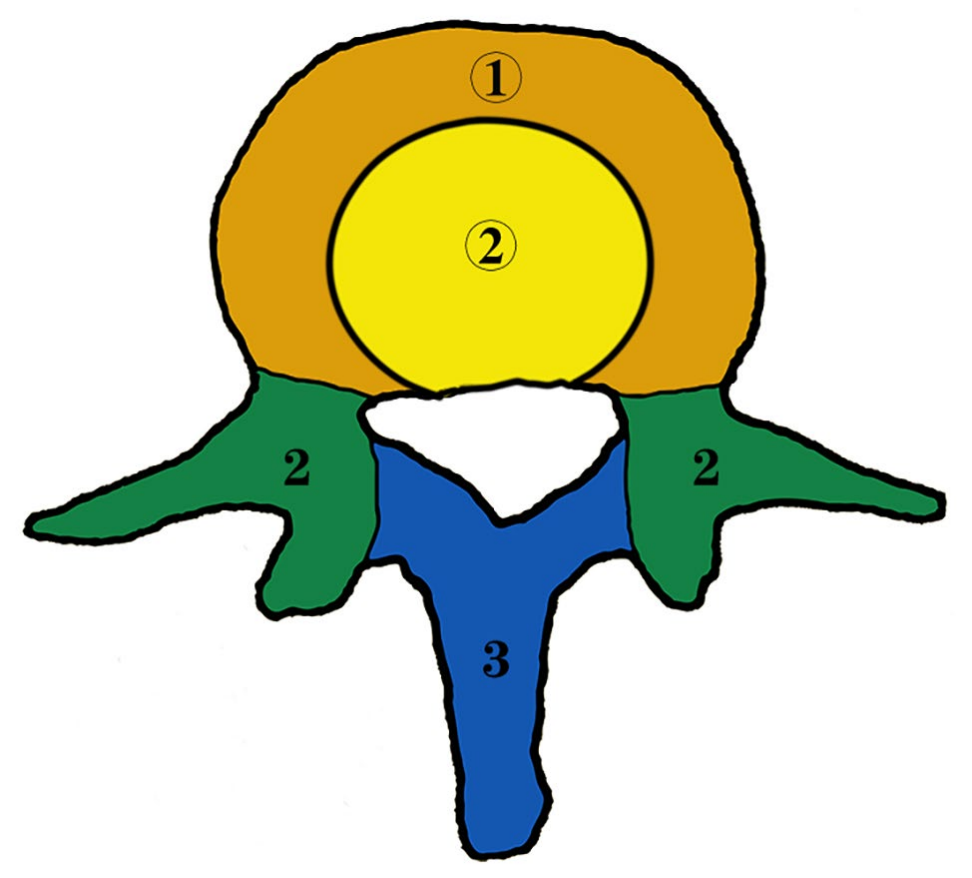
Modified vertebral zone division in transverse section

According to the vertebral vascular distribution, the vertebrae was divided into three sections (Sects. 1, 2, and 3) (Fig. 1). Section 1 refers to the vertebral body, including 2/3 of the anterior vertebral body (Zone ①) and 1/3 of the posterior vertebral body (Zone ②) (Fig. 2). The anterior vertebral body (Zone ①) is supplied by the epiphyseal artery, periosteal artery, and epiphyseal reticular artery from the segmental artery. The posterior vertebral body (Zone ②) is supplied by the nutrient artery from the anterior branch of the spinal canal artery, which derives from the spinal branch of the segmental artery posterior branch. Section 2 consists of the isthmus of the vertebral pedicle, vertebral pedicle, articular process, and transverse process. The posterior branch of the segmental artery sends out dorsal branches and transverse branches. Then the dorsal branch sends out superior articular, inferior articular, and interarticular branches to supply blood for the superior and inferior articular processes, pedicle isthmus, and vertebral pedicle. The transverse branch sends out the anterior transverse process artery and supplies blood for the transverse process. Section 3 is composed of the vertebral plate and spinous process. The posterior branch of the segmental artery sends out spinal branches and then forms posterior branches of the vertebral canal, which supplies blood for the vertebral plate and spinous process.

### Vertebral classification of metastatic spinal malignancies

**Fig. 3:**
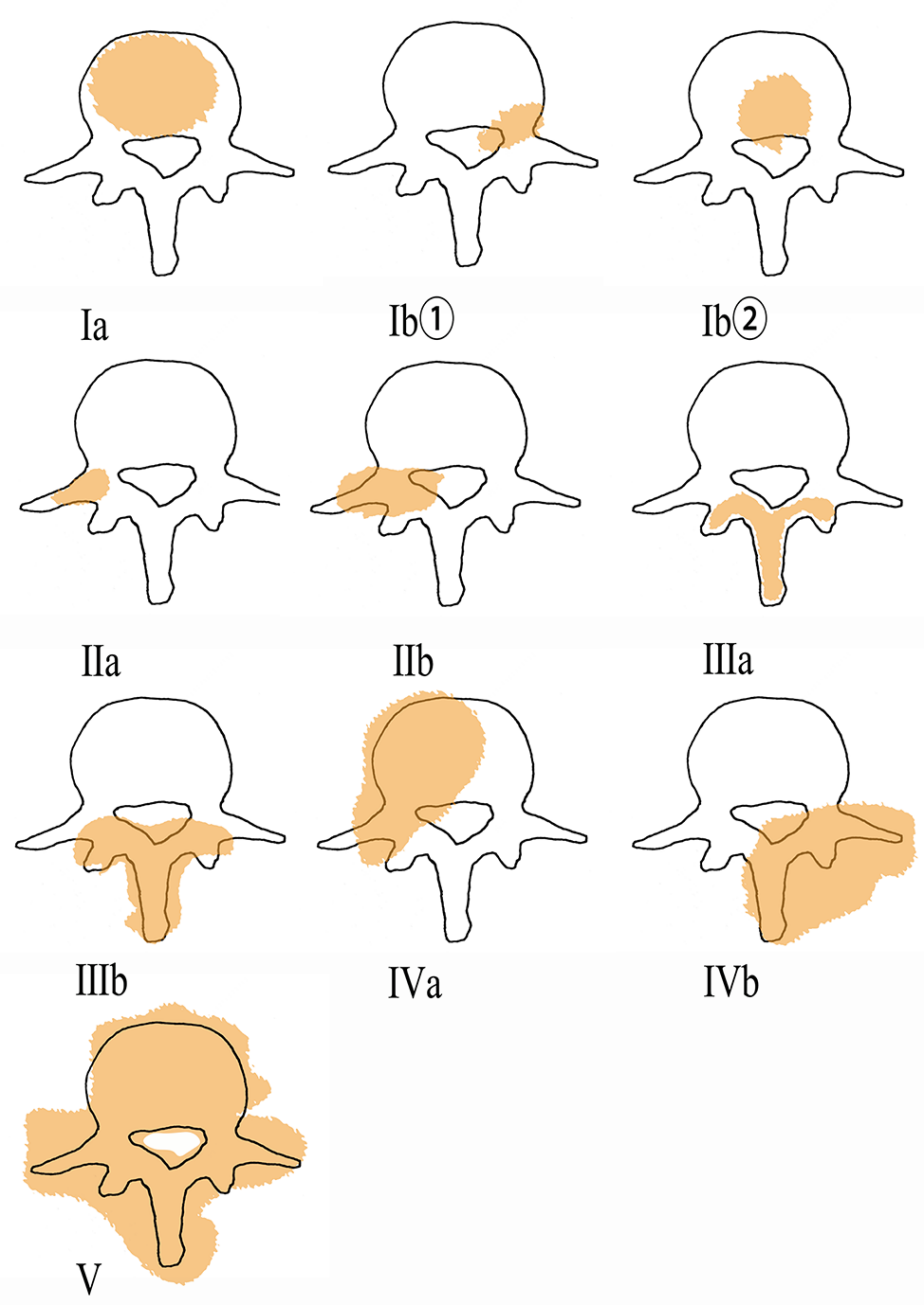
Classification of metastatic spinal malignancies

According to the vascular distribution, stability of vertebrae, and degree of nerve compression, metastatic spinal malignancies can be classified into five types. Type I refers to tumors mainly located in the vertebral body, consisting of Ia (tumors localized in the vertebral body) and Ib (tumors breaking through the posterior wall of the vertebral body). As mentioned above, depending on the differences in blood supply in different regions of the vertebral body (Zone ① is mainly supplied by the segmental arteries, whereas Zone ② derives its blood supply mainly from the spinal branches), Type Ib can be further divided into Ib① and Ib②. Type II refers to the tumors located in the vertebral pedicle, superior and inferior articular processes, and transverse processes, which can be classified into type IIa (tumors localized within the bone cortex) and type IIb (tumors breaking into the spinal canal through the bone cortex). Type III indicates the tumors located in the vertebral plate and spinous process, including IIIa (tumors localized within the bone cortex) and IIIb (tumors breaking through the bone cortex). Type IV, a complex of types I, II, and III, includes types IVa and IVb. Type IVa is a combination of types I and II (tumors located in the lateral anterior of vertebrae), while type IVb is a composite of types II and III (tumors located in the posterolateral of vertebrae). Tumors of types I, II, and III that occur together and grow from multiple directions into the spinal canal are classified as type V (Fig. 3).

### Surgical strategy

In this study, the sites of tumors were identified based on the vascular distribution in the vertebra of metastatic spinal malignancies and preoperative imaging data. The embolization and angiography of the offending arteries that fed the tumors were performed. There are several strategies for embolization. One is the embolization of the blood supply to the vertebral arch, which requires embolization of the posterior artery emanating from the segmental artery. The whole-spine blood supply was embolized for mixed tumors affecting all parts of the vertebra. All segmental and collateral arteries from adjacent segments were embolized with 0.018-inch platinum microcoils (Tornado, Cook Inc., Bloomington, IN) at the proximal of the offending arteries. A coaxial system with a microcatheter must be used to achieve a stable catheter position to place the coil. For all patients, medium-sized (150–250 μm) PVA particles (Contour, Target Therapeutics, Freemont, CA) were injected selectively into feeding arteries to block small vessels around the tumor. The injections were given at the level of the affected vertebra and at least one level superior and inferior to it. It’s important to note that embolization was not performed on segmental arteries, which supply the spinal cord. The flow during particulate embolization was determined by roadmap and angiography. At the end of the embolization, tumor vessels were completely checked by the final series of angiography, and hemostasis was performed with the coil.

Within 24 h after embolization, cytoreductive surgery and reconstruction were performed. After exposing the surgery region, the operator alternatively scrapes the tumor tissue from the lesion area with a curette and myeloid forceps. Following the tumor tissue is scraped clean and hemostasis is effective, pedicle screws are placed in the upper and lower segments of the diseased vertebrae. Based on the vertebral classification of metastatic spinal malignancies, there were four types of surgical interventions. (1) type I, curettage and reconstruction of tumors in the anterior vertebral plate of the posterior vertebral canal; (2) type II, curettage and reconstruction of tumors in the lateral vertebral plate of the posterior vertebral canal; (3) type III, curettage and reconstruction of tumors in the posterior vertebral plate of the posterior vertebral canal; and (4) types IV and V, curettage and reconstruction of surrounding tumors in the posterior spinal canal.

### Statistical methods

Categorical data were compared using Chi-squared analysis and mean values in the different treatment groups were compared using t-tests. The SPSS software (IBM SPSS Statistics for Windows, version 24.0; IBM Corp., Armonk, NY, USA) was used for statistical analysis. p < 0.05 was considered significant difference.

## Results

### General data of patients

As shown in Table 1, a total of 86 patients were included using the established inclusion and exclusion criteria. The mean age of patients in these three groups was 64.4 ± 11.2, 59.4 ± 11.1, and 57.5 ± 10.2, respectively (p = 0.065). 40 patients were female, and 46 were male (p = 0.179). The above results indicated that there was no significant difference in demographic data, such as age and gender, among the three groups. Among the 86 patients included, the top 3 primary cancers were lung cancer (38/86, 44.1%), breast cancer (13/86, 15.1%), and renal cancer (12/86, 13.9%). In 35 individuals (40.6%), the lesion occurred in the thoracic vertebrae, whereas in the other 51 patients (59.3%), it was found in the lumbar vertebrae. No significant difference was found in the revised ESCC score and SINS score (ESCC score: p = 0.356; SINS = 11.2 ± 2.9, 12.7 ± 2.3, 11.6 ± 3.2, respectively (p = 0.260)). Moreover, there was no significant difference in the procedure options for patients who underwent tumor curettage among the three groups (p = 0.859) (Table 1).

**Table 1:** Characteristics of patients in different groups

Groups	Non-embolization Group	Embolization Group	Offending Embolization Group	*P*-Value
Number of Cases	20	24	42	
Gender				0.179
Male	12	13	21	
Female	8	11	21	
Age (Years)	64.4±11.2	59.4±11.1	57.5±10.2	0.065
SINS	11.2±2.9	12.7±2.3	11.6±3.2	0.260
ESCC Grade				0.356
1	3	1	8	
2	8	7	15	
3	9	16	19	
Primary Cancer Renal Cancer 12 Thyroid Cancer 3 Breast Cancer 13 Lung Cancer 38 Prostate Cancer 7 Hepatic Cancer 3 Colon Cancer 1 Gastric Cancer 2 Esophageal Cancer 1 Bile Duct Cancer 1 Pancreatic Cancer 1 Cervical Cancer 3 Bladder Cancer 1	2 1 12 3 1 1	6 1 4 7 2 1 1 1 1	4 2 8 19 2 1 1 1 1 2 1	
Affected Part of Vertebra				
Thoracic Vertebra	2	14	19	
Lumbar Vertebra	18	10	23	
Surgical Methods				0.859
1	4	5	7	
2	5	7	15	
3	3	6	6	
4	8	6	14	

### Clinical classification of included patients

The 86 patients included in the study were clinically categorized preoperatively based on the cross-sectional CT and MRI examinations of spinal tumors. Based on the new vertebral classification of metastatic spinal malignancies, as shown in Tables 2, and 36 patients (41.9%) whose tumors were located in type Ib, 19 patients (22.1%) with tumors in type IIb, 4 patients (4.7%) with tumors in type IIIb, 24 patients (27.9%) with tumors in type IV, and 3 patients (3.5%) with tumors in type V.

**Table 2:** Tumor location of metastatic spinal malignancies

	Tumor Location				
	Ib	IIb	IIIb	IV	V
Primary Cancer					
Renal Cancer 12	6	1	1	3	1
Thyroid Cancer 3	1	1		1	
Breast Cancer 13	5	3	1	4	
Lung Cancer 38	16	8	2	10	2
Prostate Cancer 7	4	1		2	
Hepatic Cancer 3		1		2	
Colon Cancer 1	1				
Gastric Cancer 2		1		1	
Esophageal Cancer 1				1	
Bile Duct Cancer 1		1			
Pancreatic Cancer 1	1				
Cervical Cancer 3	2	1			
Bladder Cancer 1		1			

### Sites of tumor occurrence in vertebrae and offending arterial embolization

**Fig. 4:**
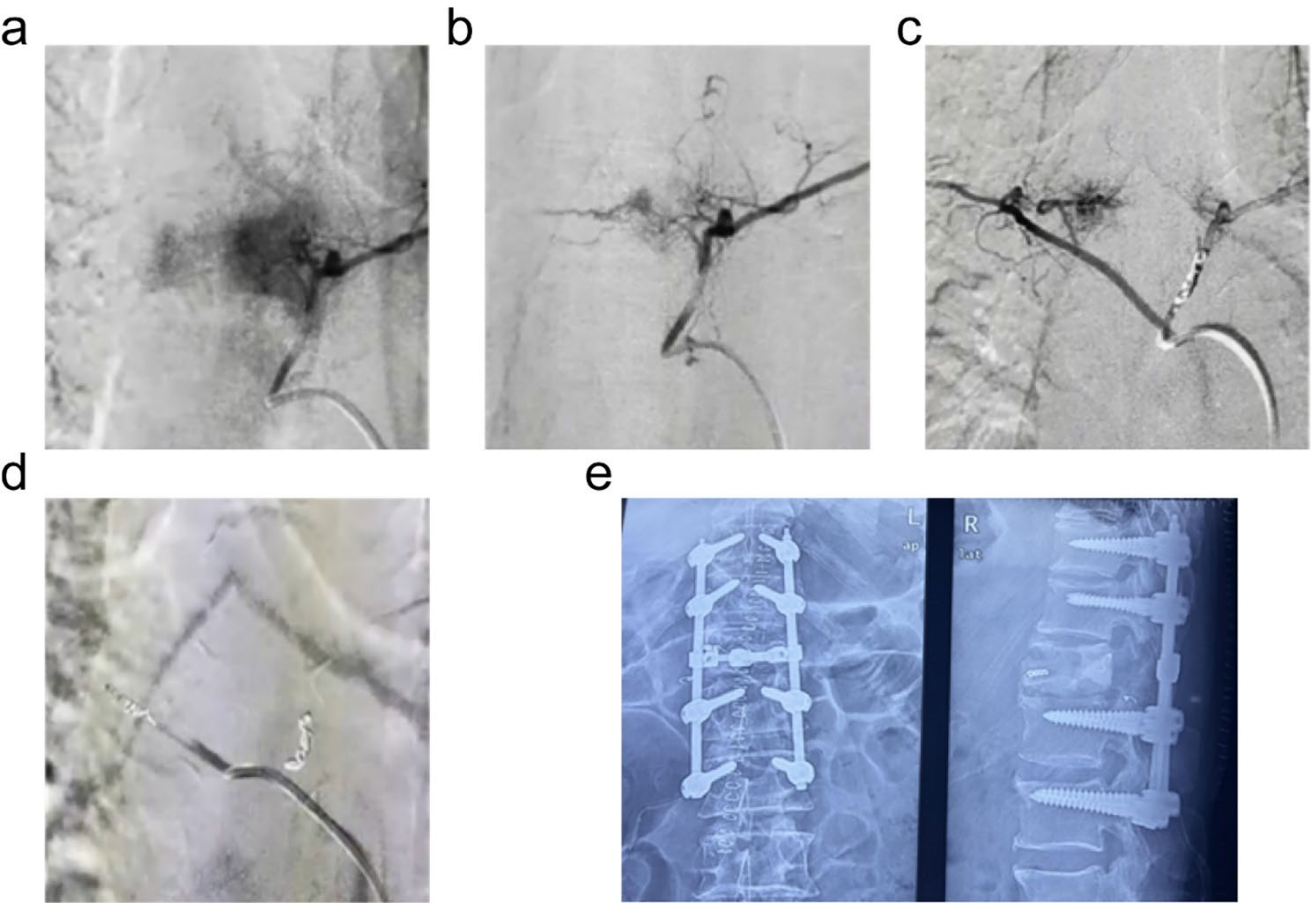
Single spinal metastasis of renal cancer. (**a**) Preoperative angiography of the left feeding artery. Intense red halo could be seen around the tumor. (**b**) The specific location of the left feeding artery was observed after the blood flow slowed down. (**c**) Preoperative angiography of the right feeding artery. Intense red halo could be seen around the tumor. (**d**) Clogged coil in its left proximity. Medium-sized (150–250 μm) PVA particles were injected selectively to block small vessels around the tumor. The embolization of branches of inferior relevant arteries was observed. (**e**) Tumor curettage was achieved and bone cement was infused into partial tumor cavity. The spinal stability was reconstructed through the posterior approach

The offending embolization group concluded that 42 patients received offending arterials by embolizing with coils and gelatin sponge particles. According to the preoperative CT and MRI examinations of spinal tumors in the transverse section, metastatic spinal malignancies were divided into three groups. The first group consisted of 20 cases (47.6%) of tumors occurring in the vertebral body and posterior vertebral arch (Ib②, IIIb), among which 11 cases were embolized through spinal branches and 9 cases through segmental arteries. In the second group, the tumor occurred in the lateral aspect of the vertebral arch (IIb) in 11 cases (26.2%), and hemostasis by arterial embolization was performed through dorsal and transverse branches on all of them. The third group consisted of 11 cases (26.2%) of mixed -type multiple tumors and tumors in the vertebral body (Ib①, IV, V), all of which were embolized through bilateral segmental arteries. The relevant details are shown in Table 3. Moreover, specific cases of intraoperative embolization are shown in Fig. 4.

**Table 3:** Sites of tumor occurrence in vertebrae and offending arterial embolization

	Tumor Location			The Location of Offending Arterial Embolization		
	Ib②, IIIb	IIb	Ib①, IV, V	Spinal Branches	Dorsal and Transverse Branches	Segmental Arteries
Primary Cancer Renal Cancer 4 Thyroid Cancer 2 Breast Cancer 8 Lung Cancer 19 Prostate Cancer 2 Liver Cancer 1 Gastric Cancer 1 Bile Duct Cancer 1 Pancreatic Cancer 1 Cervical Cancer 2 Bladder Cancer 1	2 1 3 10 1 1 1 1	1 1 2 4 1 1 1	1 3 5 1 1	1 1 2 7	1 1 2 4 1 1 1	2 4 8 1 1 1 1 1 1

### Comparison of surgical outcomes

Between the three groups, the mean bleeding volume of patients in these three groups was 4,113.9 ± 1537.0 ml, 1,476.5 ± 371.2 ml, and 836.9 ± 270.6 ml (p < 0.001). Moreover, there were also significant differences in operation time between non-embolization group (263.4 ± 33.9 min), embolization group (233.5 ± 38.0 min), and offending embolization group (184.9 ± 30.4 min) (p < 0.001). Then, significant differences were observed in the local recurrence within one year after the operation among the three groups (p = 0.006) (Table 4).

**Table 4:** Comparison of patients’ characteristics during and after surgery among groups

Surgical Methods	Non-embolization Group	Embolization Group	Offending Embolization Group	*P*-Value
Bleeding Volume (ml)	4,113.9±1537.0	1,476.5±371.2	836.9±270.6	<0.001
Operation Time (min)	263.4±33.9	233.5±38.0	184.9±30.4	<0.001
Recurrence within 1 Year				0.006
Recurrence	13	8	10	
No Recurrence	7	16	32	

## Discussion

The advancements in medication, radiotherapy, and surgical techniques have significantly enhanced the survival rate, functional status, and overall quality of life for cancer patients. Through combining chemotherapy and surgery techniques, Zhang et al. stated that patients with various stages of advanced cancer have longer survival times (P = 0.0001) and lower recurrence rates (p < 0.00001) [14]. As technology improves, surgical treatment for metastatic tumors becomes more and more essential. Similarly, the approach to treating patients with metastatic spinal malignancies has shifted regarding the role of surgery. The surgical treatment of metastatic spinal malignancies depends on several factors, including the patient’s general health and the location and number of metastases. Objective assessments are crucial in determining the appropriate surgical approach. In general, the complete removal of the lesion is frequently emphasized in radical surgery. Due to the complexity of spinal structures, radical surgery is often accompanied by greater surgical trauma, which can be overwhelming for patients with metastatic spinal malignancies who tend to present with primary tumor lesions and poor nutritional conditions [15]. As an additional form of treatment, surgical procedures that include the elimination of tumor cells, release of nerve compression, and effective fixation of the spine are progressively being utilized for patients with metastatic spinal malignancies [16]. When selecting a surgical treatment plan, it is important to consider methods that are less invasive and allow for faster postoperative recovery. This not only improves the patient’s quality of life and reduces pain, but also provides systemic benefits for their overall treatment. Therefore, a simple operational approach should be chosen [17]. In this study, the offending vessels of the diseased vertebrae can be analyzed before the operation using CT data for metastatic spinal malignancies. Arteries to be effectively embolized preoperatively can then be planned, allowing feeding vessels to be rapidly embolized and the bleeding volume during and after the operation to be effectively reduced [18].

**Fig. 5:**
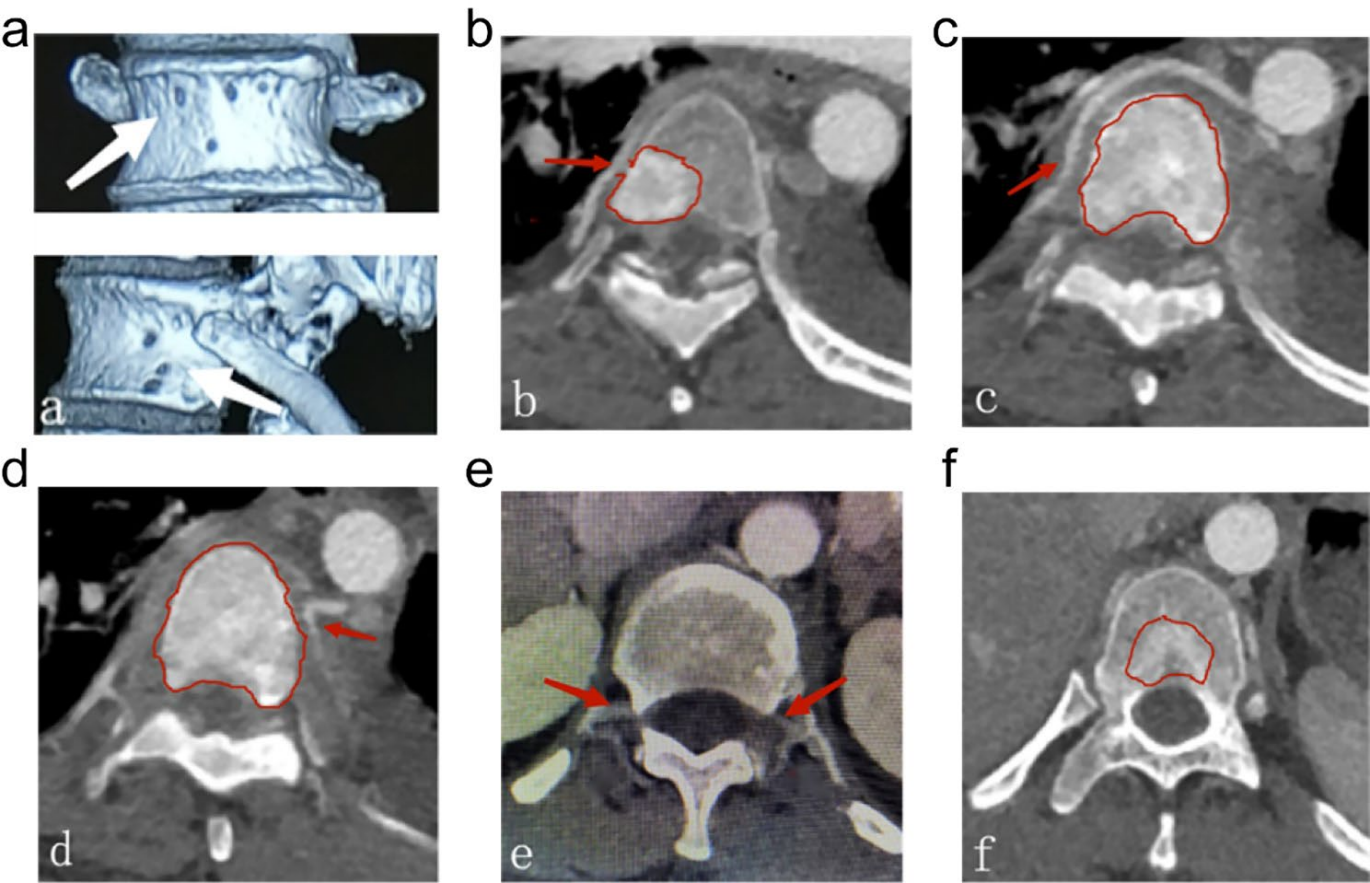
The vascular territory of the vertebral body. (**a**) Pores of vertebral arteries in nutrient arteries anterior to the vertebral body, periosteal arteries, and rete-like arteries in metaphysis (white arrow). (**b**) Enhanced CT showed the nutrient artery derived from the right segmental artery extended towards the vertebral body (red arrow) and areas of blood supply to the tumor in vertebral body (red outline). (**c**) Enhanced CT showed the nutrient artery originated from the right segmental artery was directed to the vertebral body (red arrow) and areas of blood supply to the tumor in vertebral body (red outline). (**d**) Enhanced CT showed the nutrient artery was sent from the left segmental artery to the vertebral body (red arrow) and areas of blood supply to the tumor in vertebral body (red outline). (**e**) Enhanced CT showed bilateral segmental arteries, posterior branches, spinal branches and anterior branches of vertebral canal formed the nutrient arteries that anteriorly supplied the blood flow of posterior vertebral body (red arrow). (**f**) Enhanced CT showed areas of blood supply to the tumor in the posterior vertebral body (red outline)

Tumors in the spine are distinct from those found in other areas of the body. The bone structure of the spinal column is mainly divided into the vertebral body and the vertebral arch. Compared with primary spinal malignancies, the incidence of metastatic spinal malignancies is relatively higher. According to the findings of a systematic review, the most frequently cited incidence of metastatic spinal malignancies of 30% is based on autopsy studies, and approximately 1 in 10 patients with metastatic spinal malignancies will develop metastatic epidural spinal cord compression (9.5%) or pathologic vertebral compression fractures (12.6%) [19]. In addition, this study also revealed that breast and lung cancers are the most common solid tumors that develop into metastatic malignancies of the spine, which is consistent with the results of our current investigation (lung cancer: 38/86, 44.2%, breast cancer: 13/86, 15.1%) [19]. Metastatic spinal malignancies destroy the bony structure of the vertebra, causing spinal instability [20]. The spine possesses unique physiological structures that have limited blood supply, including endplates, intervertebral discs, anterior and posterior longitudinal ligaments, and fasciae of the surrounding muscular tissues. When metastatic spinal malignancies are present, the tumor cells typically spread within the vertebra until the bony structure is destroyed, leading to pathological fractures. The tumor tissue then expands into the surrounding soft tissue and grows in an infiltrating manner, forming a tumor envelope that compresses the spinal cord and nerve roots, causing pain and paralysis. According to the location and direction of tumor growth, the tumors occurring in the vertebral bone grow into the spinal canal, towards the anterior (a), posterior (p), and lateral (t) directions of the spinal cord, respectively (Fig. 5) [21]. Some reasons for surgery are when the spinal cord is compressed within the spine or when the nerve roots outside of the spine are compressed. Depending on the particularity of the spinal structure, intralesional excision is always required to preserve important nerve and vascular structures and reduce the incidence of complications. Decompression surgery based on this special anatomy can guide the method of cytoreductive surgery [22]. The scope of cytoreductive surgery should be consistent with the location of tumor occurrence, and other adjuvant therapies should be combined postoperatively.

**Fig. 6:**
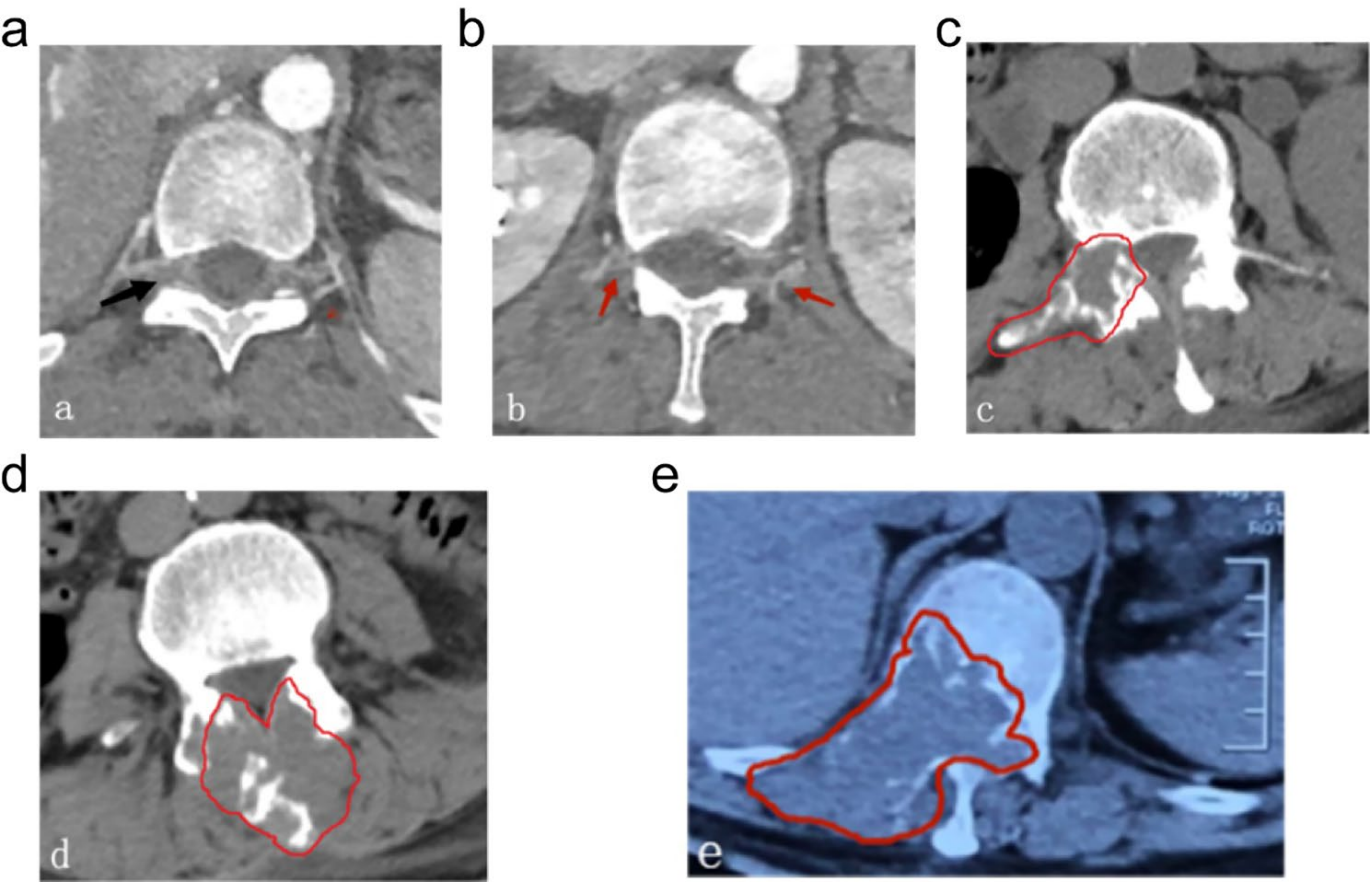
The vascular territory of the vertebral arch. (**a**) Enhanced CT showed that the right segmental artery-posterior branch-posterior branch of vertebral canal supplied blood posteriorly to the vertebral plate, spinal process, and ligamentum flavum (black arrow). (**b**) Enhanced CT showed that the bilateral segmental artery-dorsal branch-superior articular branch, inferior articular branch, and interarticular branch supplied blood to the superior and inferior articular processes, isthmus of pedicle, and pedicle. Bilateral segmental artery-transverse branch-transverse process anterior artery supplied blood to transverse process (red arrow). (**c**) CT displayed the areas of tumors in the right pedicles, transverse processes, and articular processes (red outline). (**d**) CT displayed the areas of tumors in the bilateral vertebral plates and spinal processes (red outline). (**e**) CT displayed the areas of tumors in the right vertebral body, pedicle, transverse process, joint process, and vertebral plate (red outline)

Before a surgery to treat diseased vertebrae, there are two primary ways to block blood supply. The first involves obstructing blood flow to the vertebral body by directly embolizing the main branch of the segmental artery. The second method involves obstructing blood flow to the vertebral arch by embolizing the posterior branch of the segmental artery. The segmental artery on the right side of the vertebral body travels around it, as the thoracic and abdominal aortas are situated on the left. This artery gives rise to several nutrient arteries of the anterior and lateral vertebral bodies, posterior branches of the right vertebral arch, and right intercostal arteries at the middle point. Therefore, if the right anterior 2/3 vertebral body and the right side of the vertebral arch are invaded by tumors, embolization can be performed through the right segmental artery; if lesions only occur in the right vertebral arch, embolization can be performed through the posterior branches of the right segmental artery; the left anterior 1/3 vertebral body is supplied by the left segmental artery, and the left vertebral arch is supplied by the posterior branch of the left segmental artery, so it can be embolized through the same approach after a lesion occurs [23]. Prior to the surgery, imaging data can be used to determine the zoning and classification of metastatic spinal malignancies, which serves as a preoperative guide for the location of arterial embolization. CT scans, which provide high density resolution, are advantageous in observing bone destruction, particularly in detecting early mild bone destruction and destruction of facet joints of the vertebral arch. However, it is inferior to MRI in visualizing early-stage bone marrow infiltration. Enhanced CT can show the artery course and bone morphology (Fig. 6).

Studies have shown that successful embolization of the offending vessel can significantly reduce blood supply to the tumor and minimize perioperative bleeding, as indicated by preoperative angiography of the affected vertebrae [24, 25]. It is noteworthy that a retrospective cohort study by Quraishi et al. revealed that the estimated blood loss in patients with metastatic renal cell carcinoma of the spine who underwent preoperative embolization was 1,696 ml, which is similar to the results obtained in this study (Embolization Group: 1,476.5 ± 371.2 ml) [26]. Additionally, the offending embolization group (836.9 ± 270.6 ml) in this study showed lesser surgical bleeding compared to the embolization group (1,476.5 ± 371.2 ml), which indicates that successful preoperative embolization of the responsible vessel could significantly reduce bleeding and increase patient safety during the perioperative period. Overall, by establishing appropriate vascular embolization strategies and surgical treatments based on the classification of metastatic spinal malignancies described in this study, surgeons can minimize operative time, bleeding, and local recurrence rates (12 months).

Nevertheless, our study also has certain limitations. In order to examine the efficacy of clinical surgical treatment for metastatic spinal malignancies, we conducted preoperative angiography which revealed a thinner inferior feeding artery. Moreover, the embolization materials and embolization technology, with certain limitations, cannot reach the level required by this study, and currently, only the closest origin of the above-mentioned artery can be embolized. Because of the limited number of patients and brief follow-up period, it is not feasible to establish a correlation between the current treatment approach and survival or local recurrence rate over an extended postoperative duration. Additionally, it is challenging to determine the precise therapeutic impact of preoperative arterial embolization, combined with curettage in the arterial supply region, on spinal metastases. However, multicenter clinical research with greater sample numbers can greatly ameliorate the above-mentioned limitations.

## Conclusions

In summary, the clinical classification of metastatic spinal malignancies and arterial vascular embolization procedures associated with surgical protocols developed here has scientific merit and might be useful for future research in the field. Certainly, future multicenter studies with larger sample sizes should undoubtedly be addressed to further confirm the reliability and practicality of this clinical classification of metastatic spinal malignancies.

## Data Availability

All data generated or analysed during this study are included in this published article.

## Abbreviations

IRB: Institutional Review Board
WBB: Weinstein-Boriani-Biagini
ESCC: Epidural Spinal Cord Compression
SINS: Spinal Instability Neoplastic Score
SPSS: Statistical Product and Service Solutions
CT: Computer Tomography
MRI: Magnetic Resonance Imaging
AKA: Artery of Adamkiewicz
